# Supplementary material for: Intelligent diagnosis and prediction of turbine digital electro-hydraulic control system faults: Design and experimentation
Source: PLoS One. 2023 Nov 15;18(11):e0294413. doi: 10.1371/journal.pone.0294413 (PMC10651019; doi:10.1371/journal.pone.0294413)
Supplement: Attachment — Submitted filename: Detailed Responses to Reviewer Comments(R2).docx [file pone.0294413.s002.docx]

Detailed Responses to Reviewers’ Comments

(Ref.: PONE-D-23-15933R2)

We want to thank the editors for acquiring quality reviews and the reviewers for their constructive comments. All the comments raised by the reviewers have been taken into account in our revised manuscript. Changes and additions made to address specific comments are outlined below.

## 1 Review Comments to the Author

**[Comment]**

Please use the space provided to explain your answers to the questions above. You may also include additional comments for the author, including concerns about dual publication, research ethics, or publication ethics. (Please upload your review as an attachment if it exceeds 20,000 characters)

Reviewer #2: I would like to thank the authors for revising the paper. All my comments have been addressed no further comments

Reviewer #3: The authors use the TensorFlow framework to accelerate intelligent diagnosis and prediction computing by employing an available ASIC to compute trained models. I suggest giving more insights into the embedded platform operated (Arduino, SparkFun, Board STM32F746, Himax WE-I Board, or whatever) and exposing the performance reached by this embedded platform.

It is essential to provide comprehensive information about the combination of TensorFlow with hardware, including a block diagram that clearly demonstrates the embedded platform's specific intervention in the overall system. This information can be found in lines 397-403.

**[Response for Reviewer #3]**

Thank you for your specialized questions. We provide comprehensive information about combining TensorFlow with details how the embedded platform is implemented and its specific role in the overall system.

*[Line 404-410, Page 28-29]*

*As shown in figure 14, We started with the Edge TPU, which is a smart chip with an arithmetic power of 4 TOPS. We configured the runtime environment needed for the Edge TPU. Next, we installed the Tensorflow framework, and other dependent packages. Next, the configuration files were modified and compiled. Once this was done, we put the system code on the Edge TPU and ran it to diagnose and predict system failures. This embedded platform performs faster and with less loss of accuracy than the previous control.*

*[Figure 14, Page 29.]*

Figure 14. Intervention process of the embedded platform on the system.
